# Physical and mechanical properties of wood and their geographic variations in *Larix sibirica* trees naturally grown in Mongolia

**DOI:** 10.1038/s41598-020-69781-7

**Published:** 2020-07-31

**Authors:** Bayasaa Tumenjargal, Futoshi Ishiguri, Haruna Aiso, Yusuke Takahashi, Ikumi Nezu, Yuya Takashima, Bayartsetseg Baasan, Ganbaatar Chultem, Jyunichi Ohshima, Shinso Yokota

**Affiliations:** 1 School of Agriculture, Utsunomiya University, Utsunomiya, Tochigi 321-8505 Japan; 2 Training and Research Institute of Forestry and Wood Industry, Mongolian University Science and Technology, Ulaanbaatar, 14191 Mongolia; 3 Faculty of Agricultural Production and Management, Shizuoka Professional University of Agriculture, Iwata, Shizuoka 438-0803 Japan; 4 Forest Tree Breeding Center, Forestry and Forest Products Research Institute, Hitachi, Ibaraki 319-1301 Japan

**Keywords:** Forestry, Boreal ecology, Plant breeding

## Abstract

We examined the physical and mechanical properties of wood in Siberian larch (*Larix sibirica*) trees that grow naturally in five Mongolian provenances (Khentii, Arkhangai, Zavkhan, Khuvsgul, and Selenge) and the geographic variations between them. Five trees with stem diameters of 20 to 30 cm at 1.3 m above ground were collected from each provenance. The mean values of the modulus of elasticity (MOE), modulus of rupture (MOR), compressive strength parallel to grain (CS), and shearing strength (SS) ranged from 7.03 to 9.51 GPa, 79.8 to 103.9 MPa, 46.3 to 51.1 MPa, and 10.4 to 13.0 MPa, respectively. Significant differences were found in radial and tangential shrinkage, MOE, MOR, and SS in wood among the five provenances. In addition, juvenile wood had inferior physical and mechanical properties in comparison to mature wood within and among provenances. Furthermore, there were significant differences in all examined properties, except for CS, in mature wood among the five provenances. Higher correlation coefficients were also obtained in mature wood among all mechanical properties, except for SS.

## Introduction

*Larix* species are one of the most productive forestry species in North America^1–4^, Europe^5–12^, Japan^13–24^, and China^25,26^. To date, several studies have focused on geographic variations in the physical and mechanical properties of wood in *Larix* species to determine effective wood utilization and conduct appropriate tree breeding programs^1,3–5,15,18,19,23,24^. For example, geographic variations have been found in the physical and mechanical properties of wood in *Larix kaempferi*^1,4^. A similar tendency was also found for another *Larix* species, *Larix sibirica*^5^. In addition to geographic and/or genetic variations in the physical and mechanical properties of wood, radial variations should also be considered for effective wood utilization^11,12,23–25,27^.

In general, softwood can be classified as juvenile or mature wood. Juvenile wood is characterized by a greater microfibril angle, a lower basic density, and inferior mechanical properties compared to mature wood^12–15,17,23,24,26,28,29^. In a study of 31-year-old *L. kaempferi* trees, wood density and wood mechanical properties, such as modulus of elasticity (MOE), modulus of rupture (MOR), compressive strength parallel to grain (CS), and shearing strength (SS), showed lower values in corewood (from the pith to the 15th annual ring) than in outer wood (from the 15th annual ring to the bark)^24^. Therefore, based on the nature of *Larix* species, (1) trees of these species with superior physical and mechanical properties of wood can be selected, and (2) the properties of juvenile wood should be considered for determining effective wood utilization.

In Mongolia, the total forest area is 18.3 million ha, covering 11.9% of the country^30^. Of this total area, *Larix sibirica* covers more than 70% of the forest. Since wood produced from *Larix* species is mainly utilized for structural applications, sawmilling is one of the main forest activities in Mongolia. Indeed, *L. sibirica* is the most important softwood species and could lead to economic improvement of the wood industry in Mongolia^31,32^. Thus, it is crucial to clarify the basic wood properties and lumber quality of *L. sibirica* naturally grown in Mongolia.

We have previously reported geographical variations in the growth characteristics, log properties, and lumber quality of *L. sibirica* in Mongolia^33–36^. We found significant differences in the tree height, dynamic Young’s modulus of logs, annual ring width, and bending properties of lumber among five provenances in Mongolia, although the stem diameters 1.3 m above ground were almost the same. Further, we found that MOE values increased up to 4 cm from the pith and then remained almost constant in *L. sibirica* grown in Tosontsengel, Mongolia, which suggests that juvenile wood might be present within 4 cm from the pith^27^. However, detailed information about geographical variations in the physical and mechanical properties of wood and characteristics of the juvenile wood is still limited for *L. sibirica* trees that grow naturally in Mongolia.

Accordingly, the objectives of this study were to investigate the physical and mechanical properties and their geographic variations in *L. sibirica* that grow naturally in five different provenances, which are famous *Larix* forestry sites in Mongolia. The physical properties (wood density and shrinkage) and mechanical properties (static bending properties, CS, and SS) of radial variations were investigated. In addition, differences in these properties were also compared between juvenile and mature wood.

## Materials and methods

### Materials and specimen preparation

Table 1 shows geographic and climatic information for the five provenances and the growth characteristics of the harvested trees^33–36^. A total of 25 trees with straight stems that did not have any severe damage were collected from five natural *L. sibirica* forests located in five different provenances (five trees in each forest) in Mongolia^33–36^. These five provenances are famous *Larix* forestry sites in Mongolia. In the present study, we collected sample trees with almost the same stem diameters at 1.3 m above ground level from natural forests because commercial transactions are usually conducted based on the log diameter. Thus, the age of the trees varied among provenances.

**Table 1:** Geographic and climatic information regarding the sampling stands and growth characteristics in *L. sibirica* trees used in the present study^33^.

Provenance	Latitude	Longitude	ASL (m)	Mean annual temperature (°C)	Mean integrated value of annual precipitation (mm/year)	*n*	NAR		D (cm)		TH (m)	
							Mean	SD	Mean	SD	Mean	SD
Khentii	48°51ʹN	110° 05ʹE	1,214	− 1.6	368	5	72	5	25.2	0.3	19.5	1.4
Arkhangai	47°22ʹN	101° 43ʹ E	1707	0.5	377	5	44	4	26.6	0.2	11.6	1.2
Zavkhan	48°41ʹN	98°17ʹE	1878	− 5.0	242	5	193	6	23.6	0.2	15.9	1.0
Khuvsgul	48°31ʹN	99°15ʹE	1827	− 1.8	226	5	49	5	22.5	0.3	15.1	0.7
Selenge	48°41ʹN	106°52ʹE	1,120	0.2	271	5	52	8	22.5	0.2	17.3	2.7

*ASL* above sea level, *n* number of sample trees, *NAR* number of annual rings at 1.3 m above ground level, *D* stem diameter at 1.3 m above ground level, *TH* tree height, *SD* standard deviation. Data on annual temperature and precipitation were provided by the Information and Research Institute of Meteorology, Hydrology, and Environment, Mongolia.

After harvesting the trees, logs 50 cm in length were collected from 0.8 to 1.3 m above the ground, and disks 2 cm in thickness were also collected at 1.3 m above the ground. To determine the radial variations in the physical and mechanical properties of the wood, bark-to-bark radial boards containing the pith (30 mm in thickness) were cut from the 50-cm-long logs. After air-drying in the laboratory (temperature controlled by an air conditioner, but humidity not controlled), the boards were planed to a thickness of 20 mm. They were then successively cut from the pith to the bark at 20-mm intervals in two directions to obtain specimens of air-dry density (AD) and oven-dry density (OD) (ca. 20 (R) × 20 (T) × 10 (L) mm) as well as for a shrinkage test (ca. 20 (R) × 20 (T) × 20 (L) mm), static bending test (ca. 20 (R) × 20 (T) × 320 (L) mm), and shearing test (chair shape, ca. 20 (R) × 20 (T) × 20 (L) mm). For the wood density, shrinkage, bending, and shearing tests, there were a total of 45 specimens collected from Khentii, 48 from Arkhangai, 42 from Zavkhan, 38 from Khuvsgul, and 40 from Selenge. Specimens (ca. 20 (R) × 20 (T) × 40 (L) mm) for the compressive test were obtained from the specimens used for the bending test that did not have any visual defects^22,27^. The number of specimens used for the compressive test was the same as used for the bending test.

### Physical properties of wood

Physical properties, except for cold-water extracted OD (EOD), were determined based on the Japanese Industrial Standards (JIS) Z 2101:2009^37^.

The AD and OD were calculated by dividing the air-dry weight by the volume of the specimens in the air-dry condition and the weight by volume in the oven-dry condition (105 °C). After measuring the OD, oven-dried specimens were soaked in running tap water for 2 weeks. Subsequently, the specimens were oven-dried again at 105 °C for 72 h to measure the oven-dry weight of the specimens. Cold-water extracted OD (EOD) was calculated by dividing the oven-dry weight of the extracted wood specimens by the volume of the extracted specimens determined by the method used for measuring the AD and OD.

Radial and tangential dimensions in the specimens were measured with a screw meter (MDC-25M, Mitutoyo) in air-dry and oven-dry conditions. The shrinkage in radial and tangential directions per 1% moisture content change was calculated.

### Mechanical properties of wood

Mechanical properties of wood, such as bending properties, CS, and SS, were determined according to the JIS Z 2101:2009^37^.

The static bending tests were conducted using a universal testing machine (MSC 5/500-2, Tokyo Testing Machine) with a span of 280 mm. A load was applied to the center of the tangential surface of the specimen at a load rate of 5 mm/min. The load and deflection were recorded with a personal computer to calculate the MOE and MOR.

The compressive tests were conducted using a universal testing machine (RTF-2350, A&D) with a load rate of 0.5 mm/min. The CS was calculated by dividing the maximum load by the cross-sectional area.

The SS tests were conducted using a universal testing machine (MSC 5/500-2, Tokyo Testing Machine) with a load rate of 0.5 mm/min. The SS was determined by dividing the maximum load by the plane area.

Before testing, the dimensions and weight of each specimen were measured to calculate the density at testing. In addition, the moisture content of bending and compressive test specimens and SS test specimens was measured after testing by the oven-dry method^37^. The mean moisture content was 7.5% for bending and compressive test specimens and 9.8% for SS test specimens, respectively. Thus, obtained data of MOE, MOR, CS, and SS were adjusted to those values at 12% moisture content by the changing ratio due to the 1% moisture content change (4, 2, 6, and 3% for MOE, MOR, CS, and SS, respectively) described by Ishimaru et al.^38^. In the present study, trees with different ages were used. Thus, MOE and MOR values for the 10th and 30th annual rings from the pith were estimated from the logarithmic formula fitted to the radial variation of MOE and MOR in each tree. The annual ring numbers of the specimens were estimated from the radial variations of annual ring width^33^ according to the method described in our previous reports^39–41^.

### Determination of boundary between juvenile and mature wood

Latewood tracheid lengths were measured to determine the boundary between juvenile and mature wood. Pith-to-bark strips were obtained from the 2-cm-thick disk. Small sticks of latewood were collected from each strip at every 5th annual ring, from the pith to the 20th annual ring from the pith, and also at every 10th annual ring. For Zavkhan, sticks were also collected at the 120th and 170th annual rings. The small sticks were macerated with Schulze’s solution. A total of 50 tracheids at each radial position were measured using a microprojector (V-12B, Nikon) and a digital caliper (CD-30C, Mitutoyo). The percentage of the annual increment of tracheid length was calculated using a logarithmic formula^42^. Then, the boundary between juvenile and mature wood was determined as the point of 1% annual growth of the latewood tracheid length^42^.

### Statistical analysis

Statistical analyses were conducted using R software^43^. Mean values of each physical and mechanical property were calculated by averaging the values of each radial position of harvested trees within a provenance. Due to failure to collect data during the static bending test, MOE and MOR were missing for one tree in Khentii. Using the mean values for each tree, the physical and mechanical properties among provenances (five trees in each provenance, total 25 trees) were evaluated with a one-way analysis of variance (ANOVA). Variances of populations were assumed as equal in the ANOVA test. In addition, a *t*-test was also performed to evaluate the differences in physical and mechanical properties between juvenile and mature wood, between OD and EOD, and between values of MOE and MOR at the 10th and 30th annual rings for each provenance (five trees) or all trees (25 trees from five provenances). To clarify the relationships between the measured properties, Pearson correlation coefficients were determined. Then, the test of no correlation was also applied.

## Results

### Physical and mechanical properties of wood

**Figure 1:**
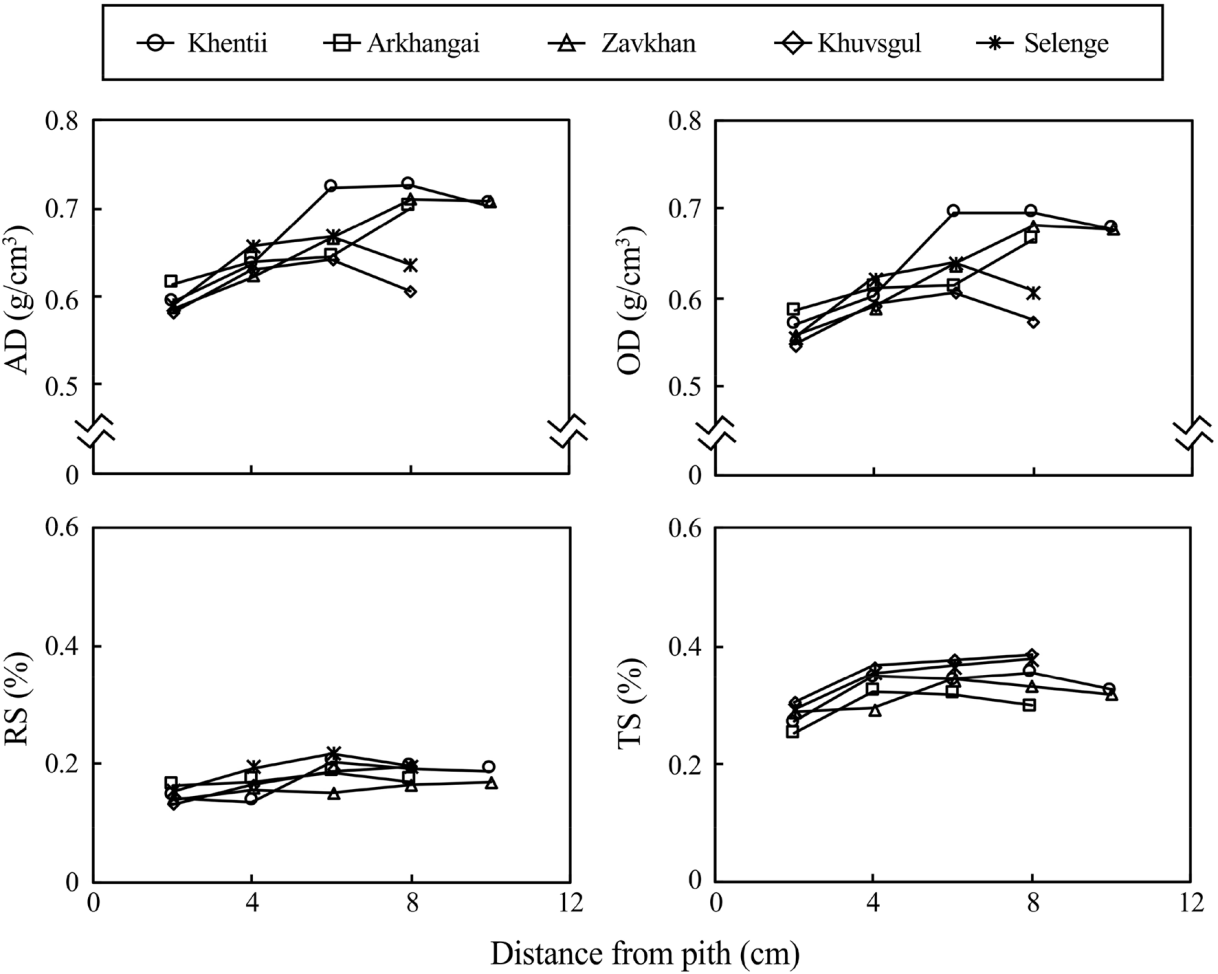
Radial variations of physical properties in *L. sibirica* obtained from different provenances in Mongolia. Each legend indicates the mean value of each radial position of five harvested trees in each stand. *AD* air-dry density, *OD* oven-dry density, *RS* shrinkage in radial direction per 1% moisture content change, *TS* shrinkage in tangential direction per 1% moisture content change.

The AD values in the five provenances ranged from 0.62 to 0.68 g/cm^3^, while the OD values ranged from 0.58 to 0.65 g/cm^3^ (Table 2). The highest values were found in Khentii and the lowest values were found in Khuvsgul for both AD and OD. Cold-water extractive content ranged from 7.3 to 16.1% in the five provenances (Table 3). Radial variations of AD and OD were similar for the five sites: slightly lower values were found near the pith compared with those at other radial positions (Fig. 1). The mean values of radial shrinkage per 1% moisture content change ranged from 0.16 to 0.19%, and tangential shrinkage per 1% moisture content change ranged from 0.30 to 0.36% among the five sites (Table 2). The shrinkage in radial direction was almost constant from the pith to the bark, whereas the shrinkage in the tangential direction increased up to 4 cm from the pith and then remained constant around 0.3 to 0.4% (Fig. 1). Significant differences among provenances were found in shrinkage and cold-water extractive content, whereas no differences were found in AD, OD, and EOD (Tables 2 and 3).

**Table 2:** Mean values and standard deviations of physical properties and tracheid length in each provenance.

Provenance	*n*	AD (g/cm^3^)		OD (g/cm^3^)		RS (%)		TS (%)		TL (mm)	
		Mean	SD	Mean	SD	Mean	SD	Mean	SD	Mean	SD
Khentii	5	0.68	0.07	0.65	0.06	0.17	0.02	0.33	0.02	2.64	0.21
Arkhangai	5	0.66	0.04	0.62	0.04	0.16	0.01	0.32	0.03	2.25	0.10
Zavkhan	5	0.65	0.05	0.61	0.05	0.17	0.02	0.30	0.02	2.40	0.06
Khuvsgul	5	0.62	0.03	0.58	0.02	0.17	0.01	0.36	0.02	2.02	0.09
Selenge	5	0.64	0.03	0.61	0.03	0.19	0.02	0.35	0.02	2.36	0.17
Total/mean	25	0.65	0.04	0.61	0.04	0.17	0.02	0.33	0.03	2.34	0.24
*F*-values		1.277		1.555		3.594		5.649		13.624	
*p*-values		0.312		0.225		0.023		0.003		0.000	

*n* number of trees, *AD* air-dry density, *OD* oven-dry density, *RS* shrinkage in radial direction per 1% moisture content change, *TS* shrinkage in tangential direction per 1% moisture content change, *TL* latewood tracheid length, *SD* standard deviation. Each value of physical properties and TL in each provenance was calculated by averaging the mean values of five trees. The mean value in each tree was calculated by averaging the values obtained from different radial positions. *F*-values and *p*-values were obtained with a one-way ANOVA among provenances.

**Table 3:** Mean values and standard deviations of oven-dry density after cold water extraction in each provenance.

Provenance	*n*	EOD (g/cm^3^)		Sig	Cold-water extractive content (%)	
		Mean	SD		Mean	SD
Khentii	5	0.56	0.05	0.043	12.3	3.3
Arkhangai	5	0.52	0.02	0.000	16.1	2.7
Zavkhan	5	0.54	0.04	0.033	11.9	3.2
Khuvsgul	5	0.53	0.02	0.004	8.0	2.7
Selenge	5	0.56	0.03	0.042	7.3	2.9
Total/mean	25	0.54	0.03	0.000	11.1	4.2
*F*-value		1. 311			7.312	
*p*-values		0.300			0.001	

*n* number of trees, *EOD* oven-dry density after cold water extraction, *SD* standard deviation, *Sig* *p*-values obtained using a *t*-test between the OD listed in Table 2 and the EOD in each provenance or all sample trees. Each value of EOD and cold-water extractive content in each provenance was calculated by averaging the mean values of five trees. The mean value in each tree was calculated by averaging the values obtained from different radial positions. *F*-values and *p*-values were obtained with a one-way ANOVA among provenances.

**Figure 2:**
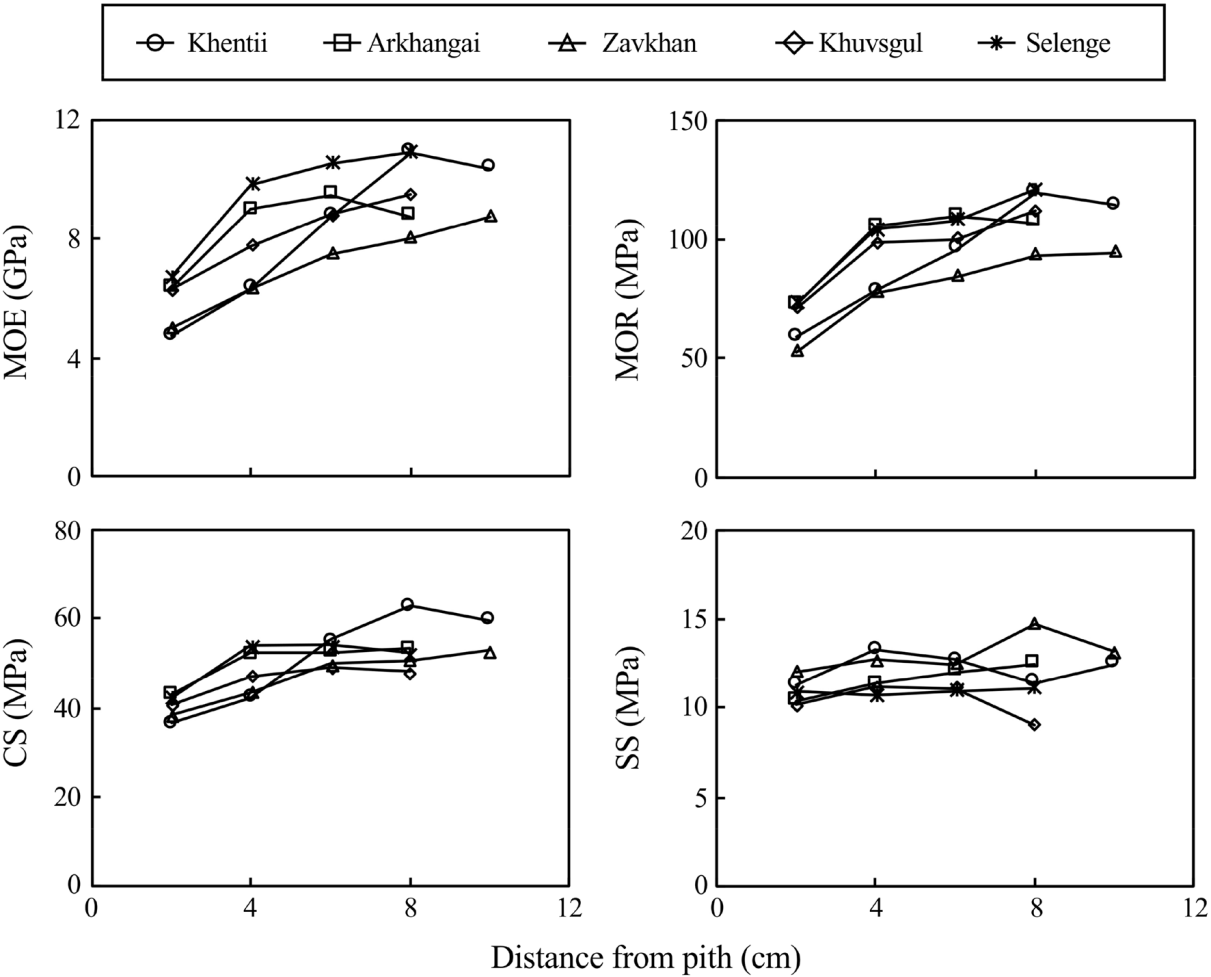
Radial variations of mechanical properties in *L. sibirica* obtained from different provenances in Mongolia. Each legend indicates the mean value of each radial position in five harvested trees in each stand. *MOE* modulus of elasticity, *MOR* modulus of rupture, *CS* compressive strength parallel to grain, *SS* shearing strength.

Table 4 shows the mean values of the mechanical properties in each provenance. The mean values of MOE, MOR, CS, and SS ranged from 7.03 to 9.51 GPa, 79.8 to 103.9 MPa, 46.3 to 51.1 MPa, and 10.4 to 13.0 MPa, respectively. The mean values of these properties in the five provenances were 8.35 GPa, 95.4 MPa, 49.0 MPa, and 11.6 MPa, respectively. The values of MOE, MOR, CS, and SS were relatively lower values around the pith, and these values increased up to 4 cm from the pith and then became constant toward the bark (Fig. 2). Significant differences among provenances were found in all mechanical properties except for CS (Table 4). Estimated MOE and MOR values at the 10th and 30th annual rings from the pith are shown in Table 5. The mean values of MOE and MOR were 6.89 GPa and 78.5 MPa at the 10th annual ring from the pith and 9.60 GPa and 108.5 MPa at the 30th annual ring from the pith, respectively. Significant differences among provenances were found in MOE and MOR at the 30th annual ring from pith.

**Table 4:** Mean values and standard deviations of mechanical properties in each provenance.

Provenance	*n*	MOE (GPa)		MOR (MPa)		CS (MPa)		SS (MPa)	
		Mean	SD	Mean	SD	Mean	SD	Mean	SD
Khentii	5 (4)	8.89	0.46	103.9	5.8	51.1	9.4	12.3	1.0
Arkhangai	5	7.03	0.50	79.8	4.9	46.8	2.7	13.0	0.3
Zavkhan	5	8.31	1.40	98.1	17.2	50.3	5.4	11.4	0.9
Khuvsgul	5	8.10	0.67	95.4	6.7	46.3	2.4	10.4	0.9
Selenge	5	9.51	0.75	101.5	8.4	50.7	2.9	11.0	1.2
Total/mean	25 (24)	8.35	1.15	95.4	12.4	49.0	5.2	11.6	1.3
*F*-values		5.912		4.475		0.965		6.432	
*p*-values		0.029		0.010		0.448		0.002	

*n* number of trees, *MOE* modulus of elasticity, *MOR* modulus of rupture, *CS* compressive strength parallel to grain, *SS* shearing strength, *SD* standard deviation. Each strength property value in each provenance was calculated by averaging the mean values of five trees. The mean value in each tree was calculated by averaging the values obtained from different radial positions. The values of the mechanical properties were adjusted to the values in the 12% moisture content condition. There were four sample trees in the static bending test in Khentii due to a failure to obtain data during the testing. *F*-values and *p*-values were obtained with a one-way ANOVA among provenances.

**Table 5:** Estimated values of MOE and MOR at the 10th and 30th annual rings from the pith.

Provenance	*n*	MOE (GPa)				Sig	MOR (MPa)				Sig
		10th		30th			10th		30th		
		Mean	SD	Mean	SD		Mean	SD	Mean	SD	
Khentii	4	6.05	1.68	11.79	1.03	0.001	76.2	14.0	132.9	17.3	0.002
Arkhangai	5	6.59	0.47	8.87	0.87	0.001	75.0	4.0	100.3	7.3	0.000
Zavkhan	5	6.78	1.67	7.80	1.38	0.324	76.3	21.3	90.6	17.0	0.275
Khuvsgul	5	6.66	0.91	9.22	1.40	0.009	77.6	11.9	109.0	15.3	0.007
Selenge	5	8.18	0.57	10.75	1.27	0.003	87.1	6.5	114.6	11.4	0.002
Total/mean	24	6.89	1.27	9.60	1.79	0.000	78.5	12.6	108.5	18.9	0.000
*F*-value		2.263		7.657			0.714		5.759		
*p*-value		0.100		0.001			0.592		0.003		

Abbreviations refer to Table 4. Sig *p*-values obtained by *t*-test between the 10th and 30th rings in a provenance or all sample trees. *F*-values and *p*-values were obtained with a one-way ANOVA among provenances. There were four sample trees in the static bending test in Khentii due to a failure to obtain data during the testing.

### Juvenile wood and mature wood

**Figure 3:**
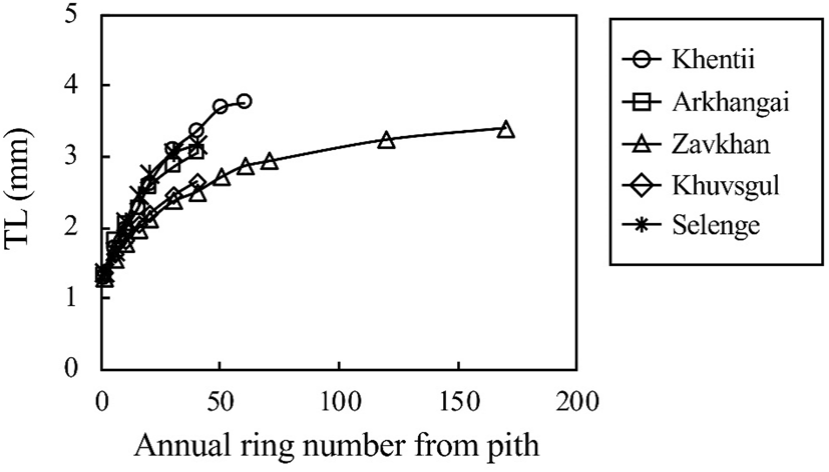
Radial variations of latewood tracheid length in *L. sibirica* obtained from different provenances in Mongolia. Each legend indicates the mean value at each radial position in five harvested trees in each stand. TL, latewood tracheid length.

The mean latewood tracheid length of the five provenances ranged from 2.02 to 2.64 mm (Table 2). The latewood tracheid length in all provenances increased up to about the 20th annual ring from the pith and then slightly increased toward the bark (Fig. 3). The boundary was determined as the point of 1% annual growth of the latewood tracheid length, according to the method described by Shiokura^42^. As shown in Table 6, the boundaries ranged from 17 to 24 annual rings from the pith among the provenances. Significant differences were found in tracheid length between the provenances (Table 2).

**Table 6:** Boundary between juvenile and mature wood based on radial variation of latewood tracheid length.

Provenance	Sample tree	Logarithmic formula	*r*^*2*^	Boundary between JW and MW (ARN from pith)
Khentii	1	*y* = 1.412 *log*_*10*_*(X)* + 1.297	0.950	22
	2	*y* = 1.000 *log*_*10*_*(X)* + 1.376	0.817	19
	3	*y* = 0.912 *log*_*10*_*(X)* + 1.388	0.821	17
	4	*y* = 0.671 *log*_*10*_*(X)* + 1.204	0.862	17
	5	*y* = 0.790 *log*_*10*_*(X)* + 1.255	0.923	18
Arkhangai	1	*y* = 1.205 *log*_*10*_*(X)* + 1.349	0.893	20
	2	*y* = 1.232 *log*_*10*_*(X)* + 1.438	0.950	20
	3	*y* = 1.491 *log*_*10*_*(X)* + 1.339	0.927	21
	4	*y* = 1.144 *log*_*10*_*(X)* + 1.339	0.918	20
	5	*y* = 1.047 *log*_*10*_*(X)* + 1.252	0.897	20
Zavkhan	1	*y* = 1.191 *log*_*10*_*(X)* + 1.399	0.870	20
	2	*y* = 1.601 *log*_*10*_*(X)* + 1.338	0.921	22
	3	*y* = 1.471 *log*_*10*_*(X)* + 1.268	0.942	22
	4	*y* = 1.508 *log*_*10*_*(X)* + 1.108	0.914	24
	5	*y* = 1.288 *log*_*10*_*(X)* + 1.351	0.958	21
Khuvsgul	1	*y* = 1.576 *log*_*10*_*(X)* + 1.399	0.875	22
	2	*y* = 1.588 *log*_*10*_*(X)* + 1.298	0.863	22
	3	*y* = 1.610 *log*_*10*_*(X)* + 1.394	0.899	21
	4	*y* = 1.652 *log*_*10*_*(X)* + 1.335	0.886	22
	5	*y* = 1.554 *log*_*10*_*(X)* + 1.336	0.948	22
Selenge	1	*y* = 1.417 *log*_*10*_*(X)* + 1.268	0.941	21
	2	*y* = 1.112 *log*_*10*_*(X)* + 1.316	0.911	20
	3	*y* = 1.133 *log*_*10*_*(X)* + 1.353	0.892	20
	4	*y* = 1.280 *log*_*10*_*(X)* + 1.451	0.896	20
	5	*y* = 0.946 *log*_*10*_*(X)* + 1.419	0.895	18
*F*-value				5.827
*p*-value				0.003

*r*^2^ coefficient of determination, *JW* juvenile wood, *MW* mature wood, *ARN* annual ring number. *F*-values and *p*-values were obtained with a one-way ANOVA for annual ring number at the boundary between JW and MW among provenances. In the logarithmic formula, *y* and *X* indicate tracheid length and annual ring number from the pith, respectively.

Mean values of the physical and mechanical properties in juvenile and mature wood are listed in Table 7. Significant differences were found in the mean values of the physical properties, tracheid length, and mechanical properties between juvenile and mature wood, except for SS. Significant among-provenance differences were found for all properties except for AD and OD in juvenile wood and CS in both juvenile and mature wood.

**Table 7:** Comparison of physical and mechanical properties between juvenile and mature wood.

Provenance	*n*	AD (g/cm^3^)				Sig	OD (g/cm^3^)				Sig	RS (%)				Sig
		JW		MW			JW		MW			JW		MW		
		Mean	SD	Mean	SD		Mean	SD	Mean	SD		Mean	SD	Mean	SD	
Khentii	5	0.63	0.06	0.73	0.07	0.038	0.60	0.05	0.70	0.07	0.028	0.14	0.02	0.20	0.03	0.006
Arkhangai	5	0.64	0.04	0.71	0.06	0.057	0.61	0.04	0.68	0.06	0.047	0.15	0.01	0.16	0.02	0.240
Zavkhan	5	0.61	0.06	0.65	0.04	0.269	0.59	0.07	0.63	0.05	0.291	0.16	0.03	0.18	0.02	0.387
Khuvsgul	5	0.61	0.04	0.62	0.02	0.707	0.58	0.04	0.59	0.02	0.745	0.15	0.01	0.20	0.02	0.004
Selenge	5	0.63	0.05	0.65	0.02	0.356	0.60	0.05	0.63	0.02	0.230	0.18	0.00	0.21	0.02	0.021
Total/mean	25	0.62	0.05	0.67	0.06	0.002	0.59	0.05	0.64	0.06	0.002	0.16	0.02	0.19	0.03	0.000
*F*-value		0.247		4.424			0.269		4.931			2.885		3.052		
*p*-value		0.908		0.010			0.894		0.006			0.049		0.041		

Provenance	*n*	TS (%)				Sig	TL (mm)				Sig	MOE (GPa)				Sig
		JW		MW			JW		MW			JW		MW		
		Mean	SD	Mean	SD		Mean	SD	Mean	SD		Mean	SD	Mean	SD	
Khentii	5 (4)	0.32	0.04	0.35	0.04	0.286	1.85	0.20	3.32	0.35	0.000	6.07	1.44	11.62	0.86	0.001
Arkhangai	5	0.31	0.02	0.32	0.06	0.445	2.01	0.07	2.92	0.15	0.000	6.61	0.32	8.32	0.84	0.003
Zavkhan	5	0.25	0.02	0.31	0.02	0.002	1.75	0.10	2.87	0.06	0.000	6.35	1.05	8.92	1.50	0.014
Khuvsgul	5	0.34	0.04	0.38	0.01	0.036	1.81	0.08	2.55	0.10	0.000	7.30	0.95	9.16	1.32	0.034
Selenge	5	0.33	0.03	0.38	0.03	0.037	2.03	0.10	3.07	0.24	0.000	8.41	0.63	10.83	1.03	0.002
Total/mean	25 (24)	0.31	0.04	0.35	0.04	0.001	1.89	0.16	2.94	0.32	0.000	6.98	1.20	9.69	1.62	0.000
*F*-value		6.913		3.241			5.177		9.397			4.872		6.594		
*p*-value		0.001		0.033			0.005		0.000			0.007		0.002		

Provenance	*n*	MOR (MPa)				Sig	CS (MPa)				Sig	SS (MPa)				Sig
		JW		MW			JW		MW			JW		MW		
		Mean	SD	Mean	SD		Mean	SD	Mean	SD		Mean	SD	Mean	SD	
Khentii	5 (4)	79.1	12.7	127.5	13.6	0.002	40.8	5.5	61.9	13.5	0.012	12.4	1.1	12.2	1.0	0.772
Arkhangai	5	75.2	3.1	92.2	7.6	0.002	45.0	2.0	52.4	6.1	0.034	12.9	0.7	13.7	1.7	0.400
Zavkhan	5	72.7	17.6	106.1	17.7	0.017	43.1	4.2	52.6	5.9	0.019	10.4	1.6	11.7	1.4	0.201
Khuvsgul	5	87.2	9.9	106.1	14.5	0.043	45.1	3.1	48.5	5.9	0.276	10.8	1.0	9.9	0.8	0.127
Selenge	5	89.5	9.2	115.4	10.2	0.003	49.0	4.0	53.0	1.7	0.074	11.0	1.0	10.7	2.1	0.780
Total/mean	25 (24)	80.8	12.5	108.7	16.6	0.000	44.6	4.5	53.7	8.3	0.000	11.5	1.4	11.6	1.9	0.809
*F*-value		2.048		4.414			2.931		2.078			4.692		4.925		
*p*-value		0.128		0.011			0.047		0.122			0.008		0.006		

Abbreviations refer to Tables 2, 3, and 4. Sig *p*-values obtained with a *t*-test between juvenile and mature wood in a provenance or in all sample trees. *F*-values and *p*-values were obtained with a one-way ANOVA among provenances. There were four sample trees in the static bending test in Khentii due to a failure to obtain data during the testing.

### Correlation among properties

**Figure 4:**
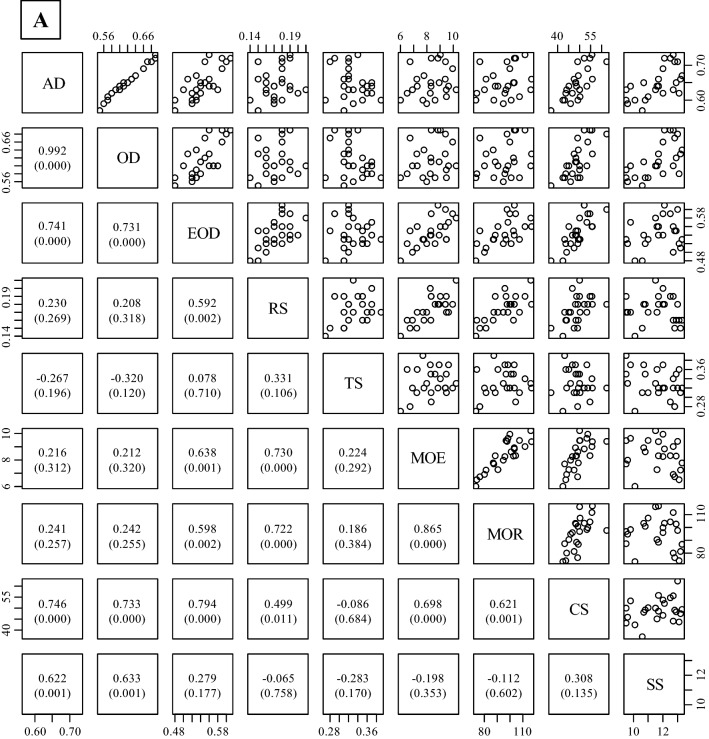

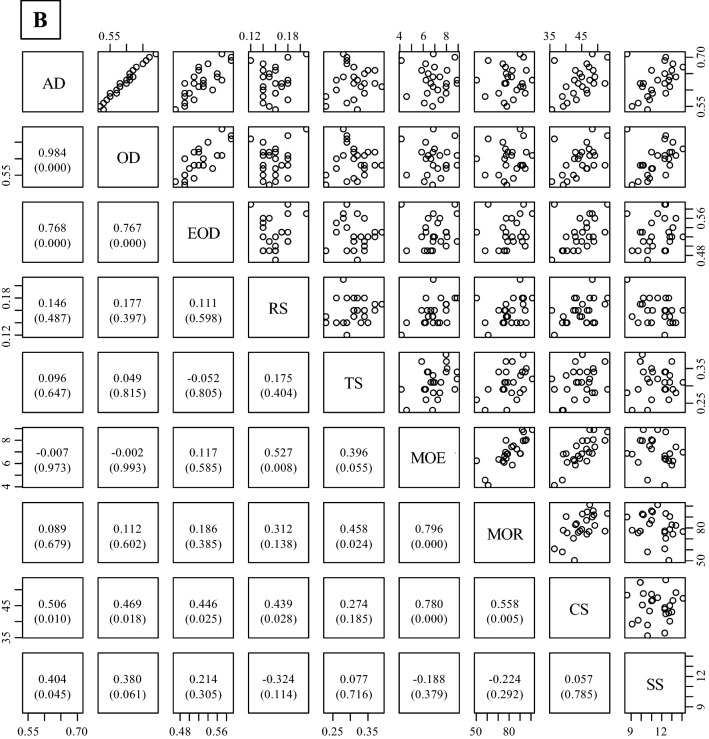

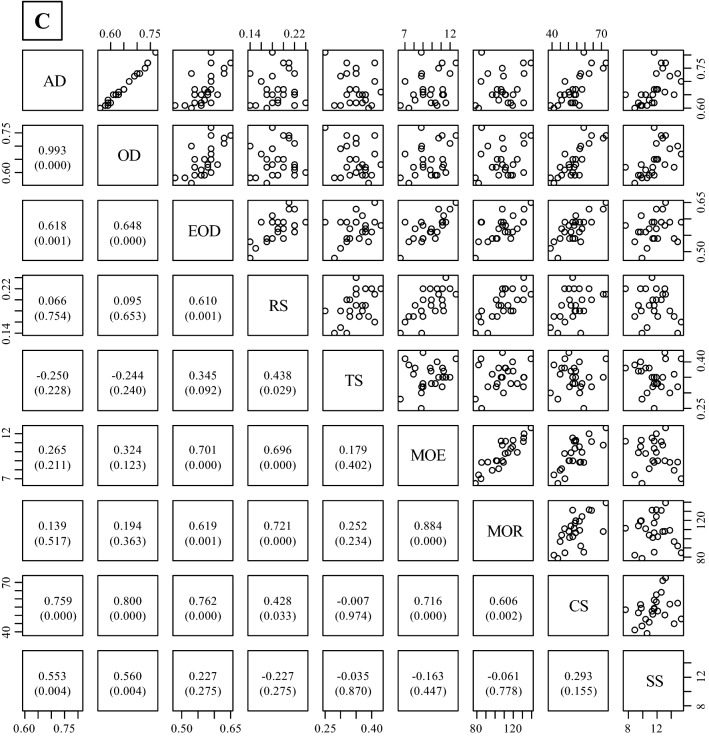
Relationships between measured properties in all wood (**A**, juvenile wood + mature wood), juvenile wood (**B**), and mature wood (**C**). Abbreviations refer to Tables 2, 3, and 4. The values in the lower diagonal indicate correlation coefficients. Values in parenthesis followed by correlation coefficients are *p*-values obtained by a test of no correlation. The number of trees = 25, except for MOE and MOR. There were four trees in the static bending test in Khentii due to a failure to obtain data during the testing. The graph was originally created by R (version 3.6.2, https://www.R-project.org/), and then *p*-values calculated by R were added in the graph.

Figure 4 shows the correlation coefficients for the physical and mechanical properties of three different wood types (juvenile wood, mature wood, and both types of wood). The mechanical properties had a strong correlation with each other for all wood types, except for SS. No significant correlation coefficients were found between RS or bending properties (MOE and MOR) and wood density (AD and OD), whereas EOD was significantly correlated with RS, MOE, or MOR in both mature wood and all wood types. However, CS and SS were significantly correlated with AD and OD in all wood types, except for SS and OD in juvenile wood.

## Discussion

### Physical and mechanical properties of wood

The AD (0.62 to 0.68 g/cm^3^) and OD (0.58 to 0.65 g/cm^3^) values obtained this study were similar with respect to basic density^33^ in the same sample trees (Table 2), whereas our results for mean AD values were relatively higher than those for *L. sibirica* reported by Ishiguri et al.^27^ and lower than those reported by Koizumi et al.^5^. Radial variations of AD and OD showed similar patterns to those reported by other researchers of *L. sibirica*^5^ and *L. kaempferi*^16^. Meanwhile, Cáceres et al.^3^ reported an influence of extractives on density in *L. kaempferi*. They found that the hot-water extractive content of *L. kaempferi* varied between 2.9 to 6.9% among 20 provenances, suggesting that actual wood density might be about 5% lower than AD. As shown in Table 3, cold-water extractive content ranged from 7.3 to 16.1%, and the mean values of EOD (0.54 g/cm^3^, Table 2) were about 10% lower values compared to OD (0.61 g/cm^3^, Table 2). These results indicated that the effect of cold- or hot-water extractives on wood density might be greater in *L. sibirica* compared to other *Larix* species.

Ishiguri et al.^27^ reported that radial shrinkage at 1% moisture content change showed almost constant values from pith to bark, whereas tangential shrinkage increased up to 4 cm from pith and then became constant at around 0.3%. The mean values and radial variation patterns examined in this study for shrinkage in both the radial and tangential directions in *L. sibirica* were similar to those of *L. sibirica* examined by Ishiguri et al.^27^.

Although the tree ages varied, the mean values of MOE, MOR, and CS of the *L. sibirica* trees in the present study (Table 4) were similar to those found in a previous study for *L. sibirica* that grow naturally in Mongolia^27^ but lower than those for *L. sibirica* that grow naturally in Russia^5^ and higher than those for *L. kaempferi* planted in Japan^22,24^. The mean SS was higher than that of *L. sibirica* planted in Finland^9^ and *L. kaempferi* planted in Japan^24^. In the radial variation, similar radial trends were found in *L. sibirica* that grow naturally in Mongolia^27^ and in *L. kaempferi* planted in Japan^22^.

Based on the obtained results, the mean values of the physical and mechanical properties of *L. sibirica* collected from five different provenances in Mongolia are similar to those of *L. sibirica* and other *Larix* species found in other countries. Thus, wood resources from *L. sibirica* harvested in Mongolia can be used for similar purposes to other *Larix* species, such as construction materials.

### Juvenile and mature wood

The boundary between juvenile and mature wood ranged from the 17th to 24th annual rings from the pith (Table 6). The results were similar to those reported for *L. kaempferi* trees^17,22,42^. However, Ishiguri et al.^27^ showed that juvenile wood might exist within 4 cm from the pith in *L. sibirica*. In the present study, the boundary was within 2 to 5 cm from the pith among the provenances, suggesting that juvenile wood formation in *L. sibirica* trees that grow naturally in Mongolia is not only affected by tree age but also by growing conditions.

We previously reported that mean values of annual ring width were 1.55, 2.47, 0.49, 1.86, and 1.74 for Khentii, Arkhangai, Zavkhan, Khuvsgul, and Selenge, respectively^33^. This result indicates that the radial growth rate was extremely slow in Zavkhan compared to other four provenances. Shiokura and Watanabe^28^ reported that suppressed radial growth in the initial stage of tree growth resulted in prolonging the juvenile wood formation period in *Picea jezoensis* and *Abies sachalinensis*. Although significant differences among provenances were also found in annual ring number from the pith in the boundary between juvenile and mature wood (Table 6), the difference in the earliest (17th) and the latest (24th) annual ring number from the pith in the boundary was only 7 years. Thus, the radial growth rate in *L. sibirica* does not have a strong effect on the cambial age at which the production of mature wood cells begins. However, further research is needed to clarify the relationship between the radial growth rate and annual ring number from the pith in the boundary between juvenile and mature wood in this species.

As shown in Table 7, significant differences between juvenile and mature wood were found in the mean values of physical properties, tracheid length, and mechanical properties, except for SS: the values of the physical and mechanical properties of juvenile wood were lower than those of mature wood. These lower values can be explained by shorter tracheid length and lower wood density. Similar results were obtained by several researchers of softwood species^17,22,24,28,29^. For example, Koizumi et al.^24^ found that, in *L. kaempferi*, the mean MOE, MOR, CS, and SS values were 8.2 GPa, 93.3 MPa, 54.0 MPa, and 11.5 MPa in juvenile wood and 9.5 GPa, 97.2 MPa, 55.1 MPa, and 11.4 MPa in mature wood, respectively. Bao et al.^25^ reported that the mechanical properties of juvenile wood were significantly lower than those of mature wood in *Larix olgenis* and *L. kaempferi*. We also found lower mechanical properties, basic density, and shorter latewood tracheid length of juvenile wood in 67-year-old *L. kaempferi*^22^. Thus, the presence of juvenile wood should be considered when utilizing wood resources of this species as construction materials requiring higher strength properties.

### Correlation among physical and mechanical properties of wood

Figure 4 shows the correlation coefficients of the physical and mechanical properties of three different wood types (all types of wood, juvenile wood, and mature wood). In general, wood density is positively related to shrinkage in the radial and tangential directions^44^. The results of this study showed significant correlations between radial shrinkage at 1% moisture content and EOD in mature wood and all wood, suggesting that EOD can predict shrinkage in the radial direction in this species. Wood density is also positively correlated with many types of mechanical properties of wood^45,46^. CS was positively correlated with all types of wood densities measured in this study. The MOE and MOR in mature wood and all wood only exhibited a significant positive correlation with EOD. These results indicate that MOE and MOR values were correlated with wood substances without extractives, and these values in juvenile wood might be related to other properties, such as microfibril angle. Luostarinen and Heräjärvi^10^ reported that water-soluble arabinogalactan contents were weakly correlated with SS in *L. sibirica*. SS was significantly correlated with AD, but not with EOD, suggesting that cold water-soluble extractives, such as arabinogalactan, might be affected on the SS in this species.

Based on these results, strength properties (e.g., bending properties and compressive strength) can be estimated with each other and predicted by EOD. In addition, SS might be influenced by the presence of cold water-soluble extractives, such as arabinogalactan.

### Among-provenance variations

Cáceres et al.^3^ reported that significant among-provenance differences were not found in basic and oven-dry densities, whereas hot-water extractive content was significantly affected by provenances in *L. kaempferi*. We also previously demonstrated that no significant differences among provenances were found in the basic density of *L. sibirica* naturally grown in Mongolia^33^. Although the cold-water extractive content significantly differed among provenances in this study, all examined densities, such as AD, OD, and EOD, showed no significant differences among the five provenances (Tables 2 and 3), indicating that wood density might not vary greatly among provenances. Thus, it can be concluded that genetic variations in relation to wood density might be small in *L. sibirica* trees naturally grown in Mongolia.

In half-sib families of *P. jezoensis*, *F*-values obtained by an ANOVA test for AD, MOE, and MOR among families gradually decreased from juvenile to mature wood^47^. In addition, Kumar et al.^48^ reported that estimates of narrow-sense heritability for MOE were generally higher in the corewood than in the outer wood in *Pinus radiata*. For *Larix* species, significant differences in wood density, CS, and SS but not in MOE and MOR were found in outer wood among 23 provenances for 31-year-old *L. kaempferi*^24^. Thus, genetic variations in the physical and mechanical properties of juvenile wood were higher than in mature wood in many softwood species. Significant differences were also found in most of the mechanical properties among provenances, except for CS (Table 4). In addition, significant differences were found in all examined physical and mechanical properties except for CS in mature wood among the five provenances, while no differences were found in juvenile wood for many properties (Table 7). Similar results were obtained in estimated MOE and MOR values at the 10th and 30th annual rings from the pith: no significant among-provenance variations were found in MOE and MOR at the 10th annual ring from the pith, but significant differences were found in the 30th annual ring from the pith (Table 5). Although the environmental conditions in the five provenances were not the same, the genetic variations in physical and mechanical properties among provenances were large in mature wood compared to juvenile wood for *L. sibirica* grown naturally in Mongolia. Further research is needed to clarify the genetic factors of the physical and mechanical properties of wood in *L. sibirica*.

Based on the results, there are significant among-provenance differences in the physical and mechanical properties of wood, especially in mature wood, in *L. sibirica* grown naturally in Mongolia. The physical and mechanical properties of wood in this species, especially in mature wood, can be improved by establishing tree breeding programs: families or clones with higher mechanical properties can be produced to achieve sustainable forestry in Mongolia.

## Conclusions

This study examined physical and mechanical properties of wood and their geographic variations in *L. sibirica* trees that grow naturally in Mongolia. Significant differences were found in RS, TS, tracheid length, MOE, MOR, and SS among five provenances. Differences in wood densities, such as AD, OD, and EOD, among the provenances were not significant. However, a significant difference was found in cold-water extractives between the provenances. The results show that the physical and mechanical properties of wood in this species can be improved by establishing appropriate tree breeding programs. In addition, the physical and mechanical properties of wood varied significantly between juvenile and mature wood. Based on the results, it is suggested that the identification of juvenile and mature wood is important for utilizing the wood of this species. In addition, identifying the extracted wood density in *L. sibirica* is also important for the practical use of the wood of this species.

## Data Availability

The data sets used in the present study can be obtained upon request from the corresponding author.
